# A Hybrid Optimization Design Method Based on TOA and GD for Improving the Diffuse Reflection Uniformity of Acoustic Metasurfaces

**DOI:** 10.3390/ma18112562

**Published:** 2025-05-30

**Authors:** Junxia Ma, Zhifeng Zhang, Yangyang Chu

**Affiliations:** College of Software Engineering, Zhengzhou University of Light Industry, Zhengzhou 450001, China; jxma@zzuli.edu.cn (J.M.); zhangzhifeng@zzuli.edu.cn (Z.Z.)

**Keywords:** acoustic metasurfaces, gradient descent, Tornado Optimization Algorithm, standard deviation

## Abstract

Acoustic metasurfaces play a key role in building acoustics, noise control, and acoustic cloaking by regulating the acoustic wave scattering characteristics through subwavelength structures. The design of diffusely reflecting metasurfaces aims to achieve a uniform distribution of a scattered field, which is essentially a high-dimensional nonconvex optimization problem that needs to balance the computational efficiency in the synergistic optimization of the spatial arrangement of cells and the angular response. In traditional methods, a heuristic algorithm is prone to local optimization, and it is difficult to balance the global search and local adjustment. And full-wave simulation is time consuming and seriously restricts the design efficiency. Therefore, the hybrid tornado-gradient descent optimization algorithm (VDGD) is proposed in this paper. It uses a two-stage collaborative optimization approach to refine the reflection angle distribution of acoustic metasurfaces, thereby enhancing the uniformity of the diffuse acoustic field. The Tornado Optimization Algorithm (TOA) was initially employed to introduce global perturbations to the randomly initialized design. Local optimization can be avoided by gradually decreasing the perturbation magnitude, which reduces the standard deviation of the sound field from about 5.81 dB to about 4.07 dB. Then, the gradient descent is used for local fine adjustment to further reduce the standard deviation to about 1.91 dB. Experimental results show that the VDGD algorithm outperforms the seven classical and up-to-date optimization algorithms in improving scattering uniformity. This method achieves an effective balance between global search and local fine tuning, providing an efficient and flexible optimization strategy for metasurface design, which can bring application support for intelligent acoustic devices and sound field regulation.

## 1. Introduction

As an artificial structure with sub-wavelength scale, the acoustic metasurface can accurately control the acoustic propagation behavior through discrete phase modulation. The design principle is to discretize the continuous phase gradient into periodically arranged functional units and to then use the phase difference between the units to construct the equivalent refractive index gradient, thus breaking the limitations of Snell’s law and realizing broadband acoustic wave reflection deflection [1], unidirectional transmission [2], and ultrasonic focusing [3].

Veselago [4] first theoretically elucidated the fundamentals of negative refractive index materials, laying a theoretical foundation for artificially modulating fluctuating physical fields. Cummer [5] subsequently provided a comprehensive review of acoustic metamaterials, systematically categorizing recent advances in zero/negative refraction, cloaking, and wavefront engineering. Addressing broadband absorption requirements, Trematerra and Bevilacqua [6] achieved multi-band attenuation through honeycomb-porous hybrid structures, and their geometric parameter sensitivity analysis informed the resonator design. Then, the feasibility of attaining broadband acoustic functionality through multiple resonant units was demonstrated by the local multi-resonant metamaterials of related multi-resonant scatterers [7]. Li et al. [8] applied the generalized Snell’s law to acoustics for the first time. By combining the folded acoustic channel with the phase gradient design, the phase gradient in the interface direction was constructed to realize the acoustic functions, such as the plane-to-surface wave conversion, anomalous deflection, and focusing, etc. It also experimentally verified the feasibility of it [9]. Subsequently, Tian et al. [10] designed a frequency-independent metasurface based on a pentamode material, which is capable of simultaneous generation of Bessel beams and conversion of multiple wave types over a wide frequency band. Zhang et al.’s [11] bandgap engineering theory for resonant metasurfaces establishes a multiscale design paradigm for broadband acoustic devices. Pioneering work by Li et al. [12] introduced geometric asymmetry-induced Willis coupling, creating non-reciprocal wave propagation mechanisms that expand metamaterial functionality. Notably, the emergence of field-responsive metamaterials [13,14,15] (electro/magneto/thermal-tunable systems) offers crucial technical references for developing our tunable gradient metasurfaces. These studies laid the foundation for acoustic metasurface design and led to the expansion of functionality.

The traditional design process of metasurfaces usually relies on repeated iterations of simulation and optimization, which is a typical “trial-and-error” design method. As the study of acoustic hypersurfaces has continued, the limitations of localized cell design of hypersurfaces through the generalized Snell’s law have been gradually recognized, and researchers have proposed a variety of new design ideas. Level set topology optimization methods based on two-scale homogenization [16], machine learning methods [17], global optimization strategies [18], and coded acoustic hypersurfaces [19] have been studied.

On the other hand, various kinds of numerical and intelligent algorithms have been continuously applied to acoustic metasurface optimization. Gao et al. [20] optimized the geometry of the labyrinth absorber by genetic algorithm (GA) to achieve ultra-thin and wide bandwidth absorption. Mohamadjavad [21] used GA to optimize the structural parameters related to mass configuration to achieve maximum acoustic attenuation and to widen the band gap. Gong [22] used Taboo GA to optimize the arrangement order of structural units controlling different foci in acoustic metasurfaces and achieved a good focusing effect after optimization. Yan et al. [23] used particle swarm optimization (PSO) to optimize the parameters of honeycomb microperforated plates to obtain wide-angle and low-frequency acoustic absorption characteristics. Shah et al. [24] used the dual-depth Q-learning network and the depth deterministic strategy gradient algorithm to optimize the metamaterials design. Compared with the advanced optimization algorithm using the fmincon function, the design of the reinforcement learning algorithm performed well. Peng [25] used Kriging and NSGA-II algorithms to achieve multi-objective sound insulation optimization of low-frequency membrane-type metasurfaces and successfully designed acoustic metamaterial structures with specific sound insulation frequencies and bandwidths. Liu [26] et al. proposed a deep neural network model based on hyperparameter optimization to predict the acoustic performance of a multi-dimensional Helmholtz resonator. Zhao et al. [27] critically evaluated machine learning applications in metamaterial inverse design, with their feature mapping models directly informing our algorithmic architecture. The data-driven optimization strategy by Wang et al. [28] complements our hybrid optimization framework in parameter sensitivity analysis. Liang et al. [29] proposed a demand-driven inverse design and optimization method based on the integration of multiple algorithms for ultra-thin broadband acoustic absorptive metamaterials, which accomplished the fast and precise customization of ultra-thin broadband acoustic absorptive properties and provided a highly efficient solution for real-time/on-demand metamaterial design. Zhou et al. [30] proposed a continuous parameterization method based on level set functions to optimize the cell distribution of hypersurface arrays without gradient information for multi-objective optimization of bandpass reflection and diffuse scattering. Lee et al. [31] demonstrated a 20% enhancement in passband absorption through genetically algorithm-optimized functionally graded metamaterials, validating the need for multi-unit synergy. Morris [18] et al. used genetic algorithms to optimize the geometric variables of metamaterials, mapping geometric parameters to equivalent mass/stiffness parameters through a reduced-order model, significantly reducing computational time, and achieving efficient optimization of high-dimensional metamaterial design.

Although significant progress has been made in enhancing specific functions of metasurfaces (e.g., focusing, acoustic absorption, or unidirectional transmission), there are still few system optimizations focusing on the uniformity of diffuse reflection; in addition, there is a lack of integrated evaluation and efficient solutions of uniformity indicators, such as scattering standard deviation or mean square error, as well as a lack of analysis on the sensitivity of the initial layout and robustness of the algorithm. Diffusers can be used to propagate reflected waves in all directions, reducing the intensity of unwanted specular reflections and echoes. It can improve the reliability of underwater communication and sonar systems to optimize underwater acoustic stealth technology. To avoid the use of randomly distributed or geometrically reflective metasurfaces, a combined optimization framework of TOA and GD is proposed in this paper, which was used to optimize the uniformity of the diffuse reflection field. First, the adaptive perturbation of TOA with the Metropolis acceptance criterion was utilized to perform global search in the discrete reflection angles space, which can not only jump out of the local minimum, but also ensure rapid convergence to the global optimal region. Then, the reflection angle of each element was adjusted enumeratively by discrete gradient descent to improve the local accuracy and further reduce the standard deviation of the far-field sound pressure level. This method combines the global exploration capability and local fine-tuning performance, which can significantly improve the diffuse reflective field uniformity, as well as provides a feasible solution for acoustic metasurfaces to achieve highly robust diffuse reflective design in complex environments.

## 2. Theoretical Background and Problem Formulation

### 2.1. Metasurface Principle

**Figure 1:**
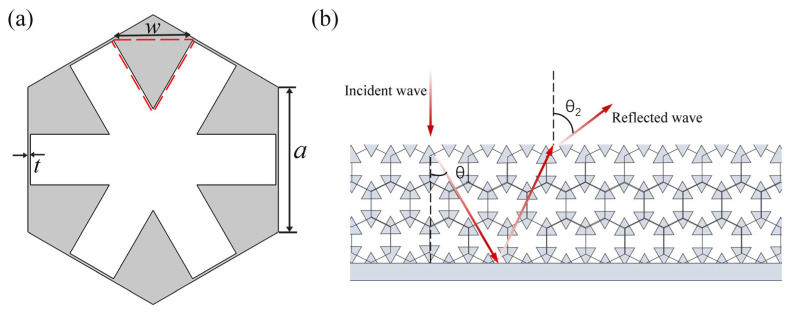
(**a**) Structure of the acoustic metamaterial. (**b**) Schematic of the acoustic wave propagation paths. The red lines and arrows indicate the transmission path of the incident acoustic wave through the metasurface.

Two-dimensional pentamode metamaterials [19,20,21] have been reported to introduce six counterweight units of the same size in a hexagonal honeycomb structure to adjust the equivalent density. As shown in Figure 1a, the structure is a regular hexagonal honeycomb lattice, consisting of six metal arms (lattice constant *a* and width *t*) and six additional counterweights (equilateral triangular regions with side length *w* surrounded by dashed lines in the figure), and these are located at the vertices of the hexagon. The gray part in Figure 1a is the metal, and the inner white skeleton is filled with air. In recent years, many researchers [22,23,24,32,33,34,35,36,37] have applied pentamode materials to underwater acoustic metasurfaces, and metal-based pentamode materials can easily match with water impedance, which has potential application value in the field of underwater acoustic control. When the acoustic wave is incident on the metasurface, the relationship between the incident wave and the transmitted wave should follow Snell’s law:

(1) $$k_{0}\left(\sin\theta_{t}-\sin\theta_{i}\right)=\frac{d\phi (x)}{dx},$$

where $\theta_{i}$ denotes the incident angle of the acoustic wave, $\theta_{t}$ denotes the refractive angle of the acoustic wave, $k_{0}$ denotes the incident wave vector, ϕ(x) denotes the phase distribution at the interface between the metasurface and the background medium, and dϕ(x)/dx denotes the phase gradient in the tangential direction (*x*-direction) of the interface. The effective parameters of the pentamode depend on the structural parameters and can be adjusted individually for phase velocity and equivalent density. The transverse gradient rate of the pentamode metamaterial is utilized instead of the gradient phase to obtain the frequency-independent Snell’s law. Thus, the transmission phase of the acoustic wave accumulation, when it is incident on the metasurface, can be expressed as follows:

(2) $$\phi (x)=lc_{0}k_{0}/c(x),$$

where c(x) denotes the gradient rate (*x*-direction), and *l* denotes the thickness of the metasurface structure (*y*-direction).

Substituting Equation (2) into Equation (1) gives the following:

(3) $$\sin\theta_{t}-\sin\theta_{i}=lc_{0}d[1/c(x)]/dx.$$

The refraction angle is determined only by the incident angle $\theta_{i}$, the thickness *l*, and the velocity gradient 1/*c*(x). The metasurface is covered on the surface of the steel plate, and two subrefractions occur at the interface when the acoustic wave is incident (the propagation path of the acoustic wave is shown in Figure 1b). When the sound wave is incident directly on the metasurface, that is, the incident angle *θ_i_* is 0, the reflection angle *θ*_2_ can be expressed as follows:

(4) $$\theta_{2}=\arcsin\left(2\times lc_{0}d\left[1/c(x)\right]/dx\right).$$

**Figure 2:**
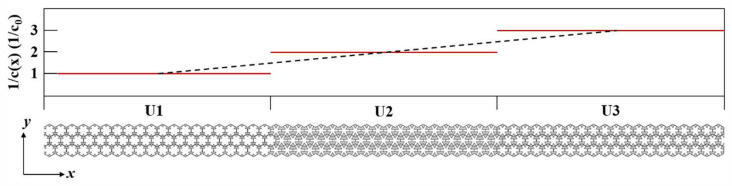
Schematic design principle of the directional reflection acoustic metasurface structure and rate gradient design. The red lines represent the reciprocal of the gradient rate corresponding to each unit.

According to Equation (4), the reflection angle *θ*_2_ of the metasurface can be adjusted by changing the rate gradient, the number of cell arrays, and the thickness of the metasurface. Eight different two-dimensional pentamode structures (denoted as U1 to U7) were utilized to construct the metasurface that matched the acoustic impedance of the background medium (water, density $\rho_{0}=1000{\;\mathrm{kg}/m}^{3}$, acoustic speed $c_{0}=1490\;m/s$). To satisfy both impedance matching and the velocity gradient, the reciprocal of the equivalent velocity $1/c_{i}$ was incremented in discrete steps $1/c_{0}$ from $1/c_{0}$ to $7/c_{0}$, while the equivalent density correspondingly varied from $\rho_{0}$ to $7\rho_{0}$ (where *ρ*_0_ = 1000 kg/m^3^ is the water density) to satisfy impedance matching. A single material cannot satisfy the wide range of requirements for both *ρ* and *c* combinations, so a two-material strategy (Ti and Pb) was used in this paper. The structural parameters of the seven pentamode metamaterial units required to design the metasurface are given in Table 1. The 3 × 18 × 3 directional reflective acoustic hypersurface (MS1) was constructed using three different cell structures, U1, U2, and U3, in Table 1. The principle of the metasurface gradient design is shown in Figure 2. As such, the directional reflection angle *θ*_MS1_ of MS1 could be obtained:

(5) $$\theta_{\mathrm{MS}1}=\arcsin\left(\frac{2\times 4ac_{0}}{18\sqrt{3}ac_{0}}\right)=\arcsin\left(\frac{4}{9\sqrt{3}}\right).$$

Therefore, the designed MS1 had a directional reflection angle *θ*_MS1_ of 14.8° for a vertically incident acoustic wave. Similarly, through using a specific combination of basic units U*_i_* with different phase velocities, it is possible to design the desired specific reflection angle. Five different directional reflection angles were designed, and all the metasurfaces used three layers, i.e., *l* = 4*a*, which were arranged as shown in Table 2.

**Figure 3:**
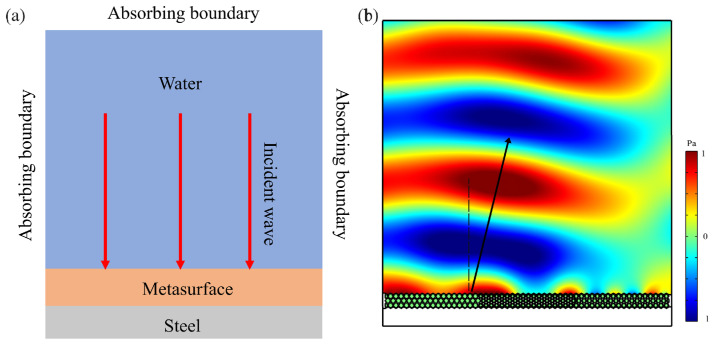
(**a**) Finite element model, the red arrows represents the incident wave. (**b**) Scattered acoustic pressure field of MS1 at 3000 Hz, the black line represents the direction of reflected sound waves.

Finite element numerical simulation was utilized to calculate its acoustic wave directional reflection characteristics to verify the feasibility of the theoretical design. Based on the hexagonal honeycomb units defined in Table 1 (e.g., U1–U7), the metasurface was constructed following the configurations in Table 2 (e.g., MS1 with a periodic arrangement of U3–U4–U5 units). The lattice constant of the unit cell was *a* = 12 mm, and the metasurface thickness was *l* = 4*a* = 48 mm, ensuring sub-wavelength operation (at *f* = 3000 Hz, the wavelength in water is λ ≈ 0.5 m, far larger than the unit dimensions). The metasurface was covered with a steel plate (thickness 0.03 m) and immersed in water. To suppress reflections, absorbing boundary conditions were applied at the outer boundaries of the water domain (labeled as “Absorbing boundary” in Figure 3a). A vertically incident plane wave (incident angle $\theta_{i}=0^{o}$, frequency *f* = 3000 Hz, pressure amplitude $P_{\mathrm{inc}}=1\;\mathrm{Pa}$) was used as the excitation. For meshing, the sub-wavelength metasurface structure was locally coarsened (cell size < λ/3) to reduce the computational cost, while the water domain was discretized using tetrahedral elements with a maximum size of λ/6 to ensure accuracy. The frequency-domain finite element solver was employed to compute the acoustic pressure distribution. As shown in Figure 3b, the simulated reflection angle of MS1 was 14.8°, which is consistent with the theoretical prediction (Equation (5)), thereby validating the design feasibility.

### 2.2. Scattering Uniformity Index and Mathematical Description

The ideal diffuse acoustic field with any point as the wave source can realize the omnidirectional uniform diffusion of acoustic energy. Moreover, its far-field acoustic pressure is uniformly distributed in [0, π], and the radiation pattern is a uniform semicircular contour. Since the acoustic pressure amplitude of plane sound waves along the propagation direction in a lossless medium remains unchanged, a diffuse acoustic field can be constructed by superimposing multiple sets of plane waves with the same amplitude to achieve equal acoustic pressure level radiation in any direction. The overall acoustic pressure distribution can be obtained by adding the acoustic pressure contributions of the metasurface units with different reflection angles:

(6) $$P(\theta )=\sum_{i=1}^{N}exp\{j[k\cdot i\cdot d\cdot cos\theta +k\cdot i\cdot d\cdot sin\theta_{t}]\},$$

where *N* represents the number of metasurface units, *k* is the wave number, *d* is the physical distance between two neighboring metasurface units, *θ* is the incidence angle, $\theta_{t}$ is the reflection angle, and *i* is the serial number of the unit, which is used to represent the *i*th unit, ranging from 1 to *N*.

To quantify the uniformity of the reflected sound field from the metasurface, the sound pressure level pattern was used in this paper.

(7) $$P_{dB}(\theta )=20log_{10}(\frac{|P(\theta )|}{P_{ref}}),$$

where ∣*P*(*θ*)∣ is the modal value of the total acoustic pressure, and $P_{ref}$ is the underwater reference acoustic pressure value of 1 μPa, which is used for normalization to quantify the uniformity of the sound field under different designs. In order to measure the uniformity of the sound field in the effective region, only the angles satisfying *P_dB_*(*θ*) > −40 dB were considered, and the average sound pressure level $\bar{P_{\mathrm{dB}}}$ was defined as follows:

(8) $$\bar{P_{\mathrm{dB}}}=\frac{1}{N^{\prime }}\sum P_{\mathrm{dB}}(\theta ),$$

where *N*′ denotes the number of sampling points within the effective region. Based on this, the standard deviation *σ* of *P*_dB_ in the effective area was used as the uniformity evaluation index to make *σ* smaller, which indicates that the sound field distribution was flatter and more uniform. The standard deviation *σ* was defined as follows:

(9) $$\sigma =\sqrt{\frac{1}{N^{\prime }}\sum {\left(P_{\mathrm{dB}}(\theta )-\bar{P_{\mathrm{dB}}}\right)}^{2}}.$$

This expression with standard deviation as the objective function provides a clear quantitative basis for the design of the metasurface reflection angle and provides a theoretical basis for the subsequent optimization of the design variables using the VDGD algorithm.

### 2.3. Modeling of the Metasurface Reflection Angle Optimization

#### 2.3.1. Design Variables and Parameters

How to configure the reflection angle of each unit to achieve a uniform scattering distribution is a key issue in the design of acoustic metasurfaces. In this paper, the metasurface reflection angle optimization problem is described as a discrete optimization problem. By regulating the direction of the acoustic pressure on the metasurface, the acoustic field exhibits a flat and uniform distribution in the target region. Therefore, the design variables of the metasurface unit are first determined, then the corresponding optimization objective function and its constraints are constructed, and, finally, the overall mathematical expression of the discrete optimization problem is determined. Let the metasurface consist of *N* cells, and let each metasurface cell *i* correspond to a selectable reflection angle *θ*_*i*_. Therefore, the design vector can be expressed as $S=[\theta_{1},\theta_{2},\ldots ,\theta_{N}]\;$, where $\theta_{i}\in \phi =\{\pm 14.8^{{}^{\circ}},\pm 29.1^{{}^{\circ}},\pm 45.2^{{}^{\circ}},\pm 60^{{}^{\circ}},\pm 80.5^{{}^{\circ}}\}$. In addition to the design variables, it also includes the speed of sound *c*, frequency *f*, and other physical parameters related to wave propagation and phase modulation.

#### 2.3.2. Optimization Objectives and Constraints

The design objective was to make the sound pressure distribution as uniform as possible over the effective angular range, i.e., to minimize the standard deviation *σ*. It can be expressed mathematically as follows:

(10) $$\min_{S}\;\;\;f(S)=\sigma \left(P_{\mathrm{dB}}(\theta ;S)\right).$$

This objective function aims to find a design vector S such that the corresponding sound pressure distribution has the smallest standard deviation to achieve uniform scattering.

In summary, the metasurface reflection angle optimization problem can be summarized as an optimization problem with discrete variable constraints, and its mathematical expression is as follows:

(11) $$\begin{array}{l} \min_{S}\;\;\;f(S)=\sigma \left(P_{\mathrm{dB}}(\theta ;S)\right)\\ s.t.\;\;\;\theta_{i}\in \{\pm 14.8^{{}^{\circ}},\pm 29.1^{{}^{\circ}},\pm 45.2^{{}^{\circ}},\pm 60^{{}^{\circ}},\pm 80.5^{{}^{\circ}}\},\;\;\;i=1,2,\ldots ,N. \end{array}$$

## 3. Algorithm Design

**Figure 4:**
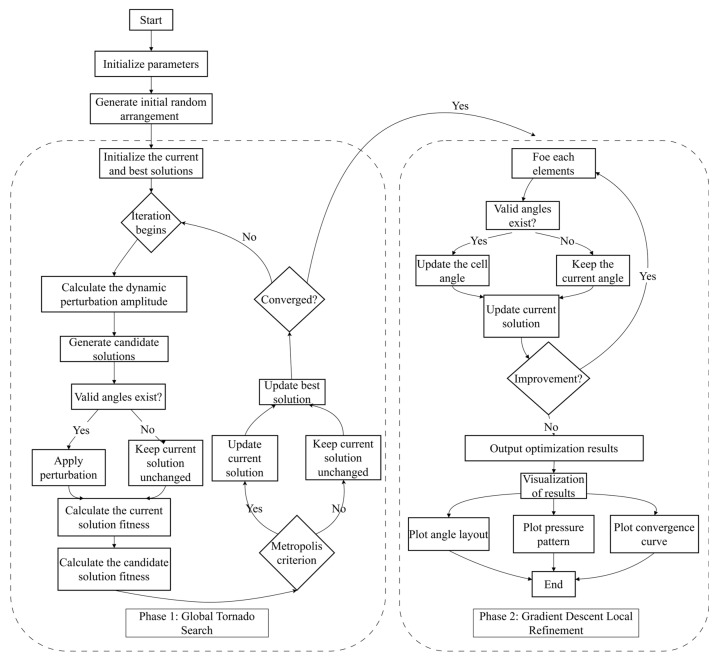
Flow chart of the metasurface optimization process.

Optimal scheduling of reflection angles for acoustic metasurfaces is essentially a high-dimensional discrete combinatorial optimization problem whose solution space grows exponentially with the number of cells. In metasurface design, especially the discrete reflection angle optimization problem, single heuristic algorithms (e.g., genetic algorithm, particle swarm optimization, etc.) are prone to local optima, while pure gradient-based approaches rely heavily on initial solution quality and struggle with discrete variables, resulting in a trade-off between optimization accuracy and efficiency. To overcome these limitations, this study proposes a hybrid optimization framework (VDGD) that integrates the TOA and discrete gradient descent, which uniquely combines global stochastic exploration with local fine tuning to address the high-dimensional non-convex optimization challenges in enhancing the diffuse reflection uniformity of acoustic metasurfaces. First, the global perturbation mechanism of TOA, which has adaptive perturbation amplitude and the Metropolis acceptance criterion, is utilized to avoid the local minima in the discrete solution space and significantly improve the global search capability. Subsequently, a discrete gradient descent is performed by enumerating finite sets of discrete reflection angles for localized refinement within globally identified regions. Through this global–local synergy, VDGD can effectively balance the search width and optimization accuracy to achieve excellent scattering uniformity and algorithmic robustness. The specific operation flow of each stage will be described in detail, and its algorithmic framework is shown in Figure 4.

In the global search phase, an initial reflection angle distribution vector $S^{0}=[\theta_{1},\theta_{2},\ldots ,\theta_{N}]\;$, whose components are from the set Φ, is randomly generated and used to initiate the search process. An objective function value, which is a metric reflecting the uniformity of the sound pressure distribution (standard deviation *σ*), is computed for the generated initial solution and serves as a benchmark for subsequent optimization. In each iteration, one or more cells are randomly selected from the current solution S, and the corresponding reflection angles are replaced with other allowable angles to generate a new candidate solution S ^∗^. This perturbation operation is similar to the “tornado” effect, where the design variables are partially randomly perturbed to broaden the solution search range while maintaining the feasibility of the solution. Then, the objective function values *σ* and *σ* ^∗^ of the current solution and candidate solutions are computed separately and updated according to the Metropolis acceptance criterion. If σ* < *σ*, the candidate solution is accepted directly; otherwise, the candidate solution is accepted with the probability $p=\exp\left(-\frac{\sigma^{*}-\sigma }{R}\right)$, where *R* = *R*_0_ exp(-iter/*N*_iter_) is the perturbation factor for stepwise decay and iter is the current iteration number. After updating the current solution, check whether the change in the objective function in 500 consecutive iterations is lower than the preset threshold *ϵ* = 0.1% or whether the maximum number of iterations is reached. If either condition is satisfied, the global search is stopped, and the current optimal solution is output as the global result. Through this global search phase, the algorithm can freely explore the solution space in a large range, effectively avoiding early local optimization and providing a better starting solution for subsequent local fine tuning.

The optimal solution *S* obtained from the global search is used as the initial input after entering the local fine-tuning phase. The purpose of this stage is to fine tune the reflection angle of each metasurface element to further reduce the objective function value. For each cell *i* in the design vector $S=[\theta_{1},\theta_{2},\ldots ,\theta_{N}]\;$, the local neighborhood $\theta_{i}=\{\theta \in \Phi |\theta \neq \theta_{i}\}$ of the unit is constructed. For each candidate angle *θ*∈$\theta_{i}$, construct a new design vector (*θ*) and compute the corresponding objective function value (*θ*). Determine the angle that minimizes the objective function value, $\theta_{i}^{\mathrm{new}}=\arg\min_{\theta \in \Theta_{i}}\sigma_{i}(\theta )$, and update the corresponding component in the current design vector. If all the candidate angles fail to reduce the objective function value, keep the original value unchanged. Check whether there still exists a cell that can bring down the objective function after completing a local traversal of all cells. Continue the iteration if available; otherwise, the local fine-tuning phase is considered to have converged and the local search is ended. Since the design variables are discrete, it is difficult to directly use continuous gradient descent. Therefore, the local fine tuning traverses the neighborhood candidate solutions by enumerating, which is equivalent to the discrete gradient descent. The method ensures a meticulous search in the neighborhood of the current solution, resulting in a continuously decreasing objective function. Combined with the better initial solution provided by the global search, local fine tuning can quickly achieve a lower objective function value than the global search to improve the overall optimization accuracy and stability.

## 4. Experimental Validation and Result Analysis

To validate the effectiveness of the VDGD algorithm, the experimental verification was systematically conducted in three stages (ensuring comprehensive and rigorous conclusions): basic diffuse reflection comparison, COMSOL simulation (In this paper, COMSOL Multiphysics 6.1 is used for numerical simulation verification.) verification, and multi-algorithm performance evaluation.

Firstly, a basic diffuse comparison was performed. To evaluate the VDGD algorithm’s capability in optimizing diffuse reflection uniformity, four scenarios were designed: without metasurface, randomly initialized metasurface, TOA-optimized metasurface, and VDGD-optimized metasurface. In this study, a one-dimensional metasurface, which contained a total of *N* = 30 metasurface units, was used as an example. The reflection angle of each unit was selected from the predefined set $\phi =\{\pm 14.8^{{}^{\circ}},\pm 29.1^{{}^{\circ}},\pm 45.2^{{}^{\circ}},\pm 60^{{}^{\circ}},\pm 80.5^{{}^{\circ}}\}$. The metasurface was used in water (sound speed *c* = 1500 m/s, frequency *f* = 3000 Hz) with the following parameters: maximum number of TOA iterations K_max_ = 500, initial perturbation amplitude R_0_ = 0.25 × N, convergence threshold ϵ = 1 × 10^−3^, maximum number of GD iterations *K*_GD_ = 100, learning rate α_GD_ = 0.05, and initial value of annealing temperature T_0_ = 2σ_0_ (where σ_0_ is the initial standard deviation). All numerical experiments were conducted on a workstation equipped with an NVIDIA GeForce GTX 4090 GPU and a 13th Gen Intel Core i7-13700 KF CPU. The optimization process was implemented on MATLAB R2023a with the Global Optimization Toolbox (v4.8) and Parallel Computing Toolbox (v7.6) for algorithm validation. Specifically, the Global Optimization Toolbox provided built-in functions for GA and PSO (e.g., ga, particleswarm, etc.), which support constraint conditions for discrete reflection angle sets. The Parallel Computing Toolbox accelerated the execution of 10 independent experiments through a multi-threaded framework (parfor loop). For the TOA and GD stages, custom scripts were developed using MATLAB’s native functions, including fmincon (nonlinear constrained optimization) and patternsearch (pattern search algorithm), thereby ensuring compatibility with discrete variable optimization and efficient global–local collaboration.

**Figure 5:**
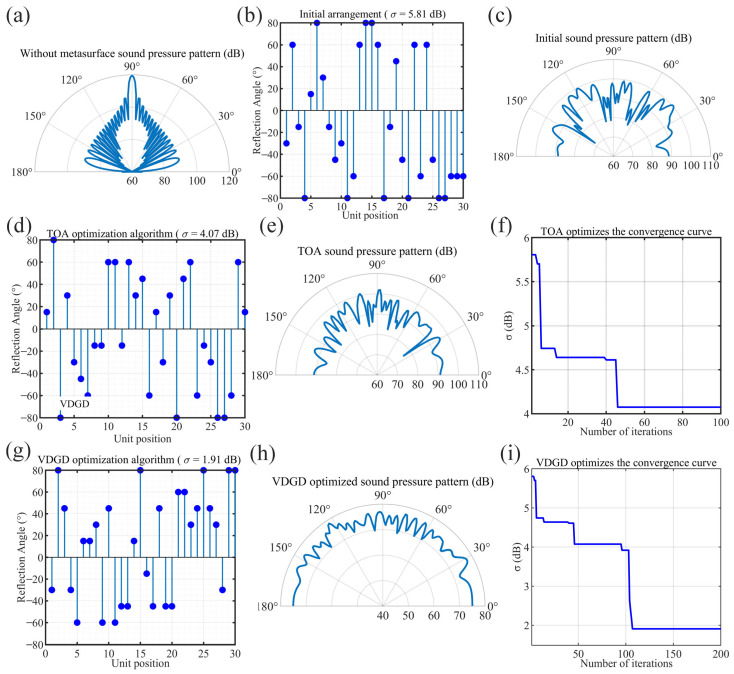
Diffuse reflective hypersurface optimization diagrams. (**a**) Sound pressure level reflection diagram without metasurface coverage. (**b**,**c**) The random initial arrangement of the metasurface units and the diffuse reflection sound pressure level diagram, respectively. (**d**–**f**) The arrangement, diffuse sound pressure diagram, and convergence curves of the metasurface units after global optimization. (**g**–**i**) The arrangement, diffuse reflection sound pressure diagram, and convergence curve of the metasurface unit after VDGD optimization. The blue dots represent the refractive angle units corresponding to different arrangements.

Figure 5a shows the far-field scattered sound pressure pattern at acoustic wave incidence in the absence of metasurface coverage, where it can be seen that the acoustic wave was reflected by the mirror image. The reflection angles of the 30 metasurface cells were first randomly generated using the random initialization method. Figure 5b shows the initial randomly arranged sequence generated before optimization, with disordered angular distribution and an initial standard deviation of 5.81 dB. Figure 5c shows the sound pressure scattering diagram corresponding to the initial arrangement, reflecting that the randomly generated initial solution had large fluctuations in the far-field sound pressure distribution and poor scattering uniformity. After the global search, the objective function value showed an obvious decreasing trend during 100 iterations. The experimental data show that the standard deviation *σ* decreased from 5.81 dB to 4.64 dB at the early stage of iteration (at about the 14th iteration), but, finally, the optimal solution obtained at the 46th iteration had its *σ* value stabilized at about 4.07 dB, which was a reduction of 29.95% (as shown in Figure 5f). Random perturbations and the Metropolis acceptance criterion were employed in this process, allowing the algorithm to jump out of the local optimum region, thus improving the scattering uniformity of the hypersurface. Comparison of Figure 5e,f shows that the acoustic wave diffuse reflectance uniformity was significantly improved after the global optimization. The local fine-tuning phase was performed after obtaining a better global solution. In this stage, the reflection angles of each metasurface cell were enumeratively fine tuned in terms of each unit for the arrangement obtained from the global search. Figure 5i shows that, after no more than 10 local adjustments in the local fine-tuning stage, the final σ value steadily decreased to about 1.91 dB, which was further reduced by 53.07% from the TOA stage. The experimental results show that the randomly initialized design performed poorly in terms of standard deviation, and the scattering distribution was significantly nonuniform. Although the global search could improve the uniformity, the ideal uniformity could not be achieved due to the lack of local fine tuning. In contrast, the combinatorial optimization algorithm fully explored the design space in the global search phase, and it then achieved detailed optimization locally, which ultimately led to it significantly outperforming all other methods in terms of the σ index. In addition, it can be observed that—through the far-field acoustic pressure pattern (Figure 5h), as well as the convergence curve (Figure 5i)—the acoustic pressure field of the combined method was smoother and more uniform, and the convergence process of the objective function was stabilized, which proves the effectiveness of the method.

**Figure 6:**
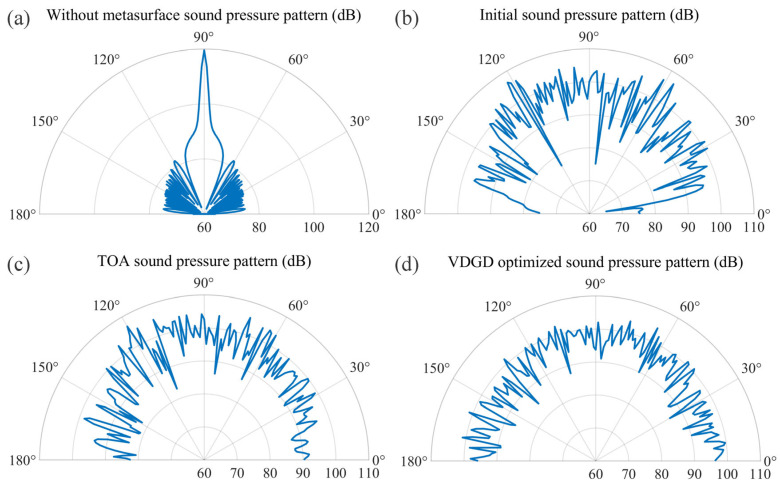
Sound pressure level scatter diagrams for the four cases of (**a**) without metasurface, (**b**) random arrangement, (**c**) TOA, and (**d**) VDGD.

Then, the COMSOL simulation verification was also performed. To further validate the proposed algorithm, the finite-element analysis software COMSOL (Multiphysics 6.1) was used to model and simulate the diffuse scattering metasurface configurations under the four scenarios mentioned above. All scenarios adopted a 30-unit one-dimensional metasurface structure modeled on a 0.03 m thick steel plate, and this was then immersed in a semicircular water domain (with a radius of 5 m, simulating an infinite field). A plane wave radiation boundary condition was applied to the water domain boundary to absorb the outgoing sound waves and eliminate reflection interference. The subwavelength-scale metasurface structure was locally refined, while the water domain was meshed with free tetrahedral elements (maximum size λ/6) to ensure accuracy. A vertically incident plane wave (3000 Hz) was applied and the far-field sound pressure level was sampled at 180 angles (0°–180°, 1° intervals), and this quantified the diffuse reflection uniformity through standard deviation (σ). The simulation results are shown in Figure 6. Without the metasurface, most of the incident sound wave’s energy was reflected in the direction perpendicular to the plane, manifesting as a main lobe in the diagram, while a small portion radiated laterally, forming side lobes. Following initial arrangement, TOA optimization, and VDGD processing, the acoustic metasurface effectively suppressed specular reflection while redirecting scattered waves significantly away from the incident direction. This transformed the incident sound wave into multiple reflected beams with reduced sound pressure levels compared to conventional plate reflections.

The VDGD-optimized metasurface exhibited a semicircular radiation profile, enabling uniform energy scattering of vertically incident plane acoustic waves. The standard deviation *σ* was calculated as the uniformity evaluation index by selecting 180 sampling points uniformly in the range of the radiation direction. The metasurface demonstrated progressively decreasing σ values of 8.41 (initial arrangement), 5.86 (TOA), and 3.85 (VDGD), indicating that the scattered sound field obtained after VDGD optimization was close to the ideal diffuse sound field, which validates the superior effectiveness of the VDGD optimization algorithm.

Additionally, to validate that the VDGD optimization model outperforms conventional design methods, it was compared with traditional approaches relying on finite element simulations. Traditional optimization methods rely on full-wave frequency-domain simulations (e.g., using COMSOL Multiphysics 6.1) in each iteration, which takes about 16 min per simulation. This is a significant computational bottleneck that severely limits large-scale iterative searches. In contrast, the proposed VDGD algorithm employs a semi-analytical framework based on far-field sound pressure superposition to quickly calculate diffuse reflection uniformity measures (e.g., standard deviation σ). This approach bypasses repetitive finite element analysis and enables the entire optimization process to be completed in less than 60 s, which is a 93.75% reduction in computational time compared to conventional FEM-driven methods.

Finally, multi-algorithm performance evaluation was conducted. To quantify the VDGD’s advantages, comparisons were conducted with seven classic algorithms (GA, PSO, Whale Optimization Algorithm (WOA), Bitter Fish Optimization Algorithm (BFO), Crowned Porcupine Optimization Algorithm (CPO), Horned Lizard Optimization Algorithm (HLOA), and the Newton–Raphson Optimization Algorithm (NRBO). All algorithms optimized the same 30-unit metasurface under identical parameters (reflection angle set Φ, *f* = 3000 Hz, water environment). Each algorithm ran 10 times with randomized initializations (population size 100, maximum iterations 100, etc.), totaling 80 experiments to mitigate initial condition bias. Optimization efficacy was quantified by the σ of the objective function, where lower σ values indicate greater stability and more successful optimization.

**Figure 7:**
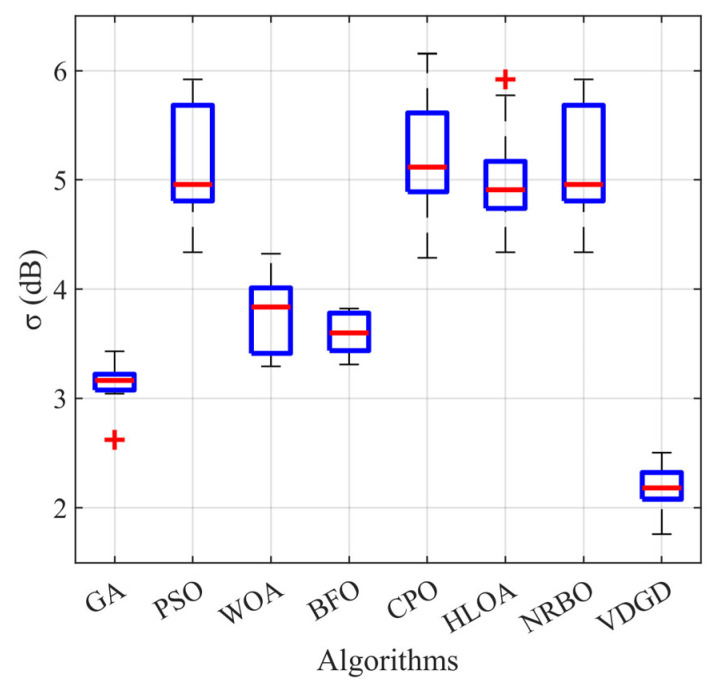
Comparison of the standard deviation boxplots for the four algorithms.

The experimental results are summarized in Table 3 and Figure 7. Table 3 reveals that the initial metasurface arrangement exhibited an average σ value of 5.63 dB under randomized initialization, indicating suboptimal energy distribution uniformity in the scattering patterns. Subsequent optimization processes yielded distinct performance across algorithms, with the final mean σ values as follows: GA at 3.14 dB, PSO at 5.11 dB, WOA at 3.77 dB, BFO at 3.59 dB, CPO at 5.23 dB, HLOA at 5.02 dB, and NRBO at 5.11 dB. In contrast, the VDGD-optimized metasurface achieved a significantly reduced mean σ value of 2.16 dB. This systematic comparison demonstrates VDGD’s superior performance in minimizing the objective function (standard deviation), exhibiting 31.21% greater stability than the second-best algorithm (GA) and outperforming conventional methods by up to 57.73% (vs. PSO).

Comprehensive algorithmic benchmarking reveals distinct performance profiles across optimization methods. The GA leverages crossover and mutation mechanisms for global solution space exploration, achieving a mean σ value of 3.14 dB, but it tends to fall into local optima under some initial conditions. PSO performed poorly after discretization with an average *σ* of 5.11 dB. While the WOA exhibited inherent advantages in continuous problem domains, its discretized implementation achieved marginally superior performance compared to PSO with a mean σ value of 3.78 dB, but there were obvious fluctuations in the solution space. The emerging BFO demonstrates notable stability (3.59 dB, 95% CI: 3.52–3.66 dB) with <5% performance variation across initial conditions. The CPO (5.23 dB) and HLOA (5.02 dB) algorithms showed high environmental sensitivity (relative standard deviation >10%), and NRBO handled the nonlinear optimization with some advantage, yet its σ rose to 5.11 dB due to the gradient collapse phenomenon that was caused by discretization. In contrast, VDGD effectively synthesized the advantages of global search and local fine tuning through the strategy of global phase exploration coupled with local phase enumeration, resulting in a reduction of σ to 2.16 dB.

Further statistical analysis using box plots on ten experimental trials visually demonstrated the performance distribution across algorithms under varying initial conditions. The VDGD algorithm exhibited superior stability metrics: its boxplot shows the lowest median value (2.18 dB) with a compact interquartile range (IQR = 0.35 dB) and an absence of outliers, indicating consistent high-quality solutions. In contrast, conventional algorithms like PSO, CPO, and NRBO displayed wider data spreads (PSO: IQR = 0.94 dB, CPO: IQR = 2.05 dB), while GA and HLOA demonstrated outlier clusters (10% and 4.7% outlier rates, respectively), signifying susceptibility to local optima entrapment and solution space oscillation. This systematic comparison reveals VDGD’s enhanced robustness and reduced dependence on initial metasurface configurations.

In conclusion, systematic benchmarking of eight optimization algorithms demonstrates that the VDGD achieves breakthrough performance in metasurface reflection angle optimization. This dual-strategy framework reduces the objective function’s standard deviation (σ) to 2.16 dB, representing a 31.21–87.77% relative reduction compared to conventional methods. The minimal differences between different initializations and the absence of outlier results for 10 trials demonstrate the excellent robustness and stability of the method, highlighting its advantages in exploring the optimization design of complex diffuse reflection acoustic metasurfaces.

## 5. Conclusions

This study designed seven water-impedance matched hexagonal honeycomb unit cells, constructing five configurable underwater acoustic metasurfaces through combinatorial arrangements of varying unit quantities. These metasurfaces enabled directional reflection control, redirecting incident waves to predetermined angular domains within the incident plane for precise backscatter manipulation. To enhance performance, we developed the VDGD hybrid optimization framework, synergistically integrating vortex-inspired global exploration with a discretized gradient descent. The algorithm implements the Metropolis criterion-guided local minima escape mechanisms followed by exhaustive micro-adjustments of individual cell reflection angles, thereby achieving optimized acoustic field uniformity.

The experimental results from 10 independent trials indicate that the proposed methodology achieved a 31.21% average reduction in sound pressure standard deviation compared with seven benchmark algorithms. The radiation pattern demonstrated enhanced conformity to the ideal semicircular distribution, significantly improving the uniformity of diffuse reflected sound fields, thereby validating the algorithm’s enhanced efficacy and robustness. The VDGD framework and optimized acoustic metasurface design proposed in this paper achieve uniform acoustic energy scattering and can be applied to engineering fields, such as concert hall and studio acoustic optimization, industrial and office noise mitigation, and underwater acoustic stealth. Future research directions include extending this methodology to higher-dimensional metasurface or volumetric array designs and incorporating multi-frequency weighted standard deviations into the objective function. These extensions aim to improve broadband uniform scattering performance and the practical applicability of metasurfaces for real-world implementations.

## Figures and Tables

**Table 1:** Geometrical parameters of the hexagonal honeycomb cells.

*a* = 12 (mm)	*w* (mm)	*t* (mm)	Materials
U1	4.336	0.254	Ti
U2	6.815	0.075	
U3	4.294	0.500	Pb
U4	5.635	0.310	
U5	6.580	0.210	
U6	7.368	0.138	
U7	8.046	0.093	

**Table 2:** Directional reflection of the metasurface parameters.

Metasurface	Unit Layout		Angle (°)
MS1	18 units each of U3–U4–U5	Thickness *l* = 4*a*	14.8
MS2	19 units each of U3–U5–U7		29.1
MS3	13 units each of U3–U5–U7		45.2
MS4	16 units each of U1–U4–U7		60.0
MS5	14 units each of U1–U4–U7		80.5

**Table 3:** The mean standard deviations of the eight different algorithms.

GA	PSO	WOA	BFO	CPO	HLOA	NRBO	VDGD
3.14	5.11	3.77	3.59	5.23	5.02	5.11	2.16

## Data Availability

The original contributions presented in this study are included in the article material. Further inquiries can be directed to the corresponding authors.
